# The Role of Brassinosteroids in Plant Cold Stress Response

**DOI:** 10.3390/life14081015

**Published:** 2024-08-15

**Authors:** Zhiqi He, Mengdi Zhou, Xiaojie Feng, Qinghua Di, Di Meng, Xianchang Yu, Yan Yan, Mintao Sun, Yansu Li

**Affiliations:** State Key Laboratory of Vegetable Biobreeding, Institute of Vegetables and Flowers, Chinese Academy of Agricultural Sciences, Beijing 100081, China; he_zhiqi00@163.com (Z.H.); 82101199108@caas.cn (M.Z.); fengxiaojie3@163.com (X.F.); 82101189108@caas.cn (Q.D.); mengdi202307@163.com (D.M.); yuxianchang@caas.cn (X.Y.); yanyan@caas.cn (Y.Y.)

**Keywords:** brassinosteroids, BZR, cold stress, signal, crosstalk

## Abstract

Temperature affects plant growth and geographical distribution. Cold stress occurs when temperatures fall below the physiologically optimal range for plants, causing permanent and irreversible damage to plant growth, development, and production. Brassinosteroids (BRs) are steroid hormones that play an important role in plant growth and various stress responses. Recent studies have shown that low temperatures affect BR biosynthesis in many plant species and that BR signaling is involved in the regulation of plant tolerance to low temperatures, both in the CBF-dependent and CBF-independent pathways. These two regulatory pathways correspond to transient and acclimation responses of low temperature, respectively. The crosstalk between BRs and other hormones is a significant factor in low-temperature tolerance. We provide an overview of recent developments in our knowledge of BRs’ function in plant responses to cold stress and how they interact with other plant hormones in this review.

## 1. Introduction

Temperature is one of the most important environmental factors that limit the geographical distribution of plant species [1,2], and it affects plant growth, productivity, and survival [3]. Cold stress includes chilling stress (0–15 °C) and freezing stress (<0 °C) [4]. Low temperatures have deleterious effects on all stages of plant development, i.e., nutritional and reproductive stages, showing signs of damage such as yellowing, wilting, and stunted growth, leading to senescence or even death [5]. Plants reprogram hormone signal transduction and change cell membrane fluidity under cold stress, which alleviates excessive accumulation of reactive oxygen species (ROS) and damage to photosynthesis at low temperatures [6]. However, plants acquire cold tolerance through cold stress acclimation and show improved freezing tolerance after exposure to non-freezing low temperatures. Complex molecular and physiological changes occur during cold acclimation, including transcriptional, translational, and metabolic changes that increase or decrease the levels of specific proteins, metabolites, and plant hormones, enabling plants to adapt to cold stress [7].

Brassinosteroids (BRs) are steroid hormones created by several hydroxylation and oxidation steps from the bulk sterol campesterol [8]. To date, a total of 81 natural BR species have been isolated from various plants, among which the most bioactive BRs are brassinolide, 24-epibrassinolide, and 28-homobrassinolide [9]. Numerous plant growth and development activities, including cell elongation, cell division, photomorphogenesis, xylem differentiation, reproduction, and response to biotic and abiotic stresses, are regulated by BRs and their derivatives [10]. BRs can regulate plant root elongation as well as promote plant growth by influencing cell division and elongation [11]. Mutations in genes associated with BR synthesis and signaling pathways lead to severe impairment of plant growth and development, resulting in severe dwarfing [12]. BRs also play a regulatory role in reproductive growth, with high content levels in seeds, pollen, and fruit, and BRs regulate floral transition, anther development, and pollen grain maturation [13]. BRs can control chlorophyllase activity to regulate chlorophyll (ChI) molecules, regulating photosynthesis in plants [14,15]. BRs respond to cold stress by regulating gene expression on multiple signaling pathways. These include the ICE-CBF-COR pathway and the non-CBF-dependent pathway, which correspond to BR-regulated transient cold stress and cold stress acclimation, respectively. BZR1-mediated transcriptional regulation is an important factor in BR-regulated cold resistance in plants, which involves the transcriptional regulation of several cold-resistant genes and interacts with multiple transcription factors to increase cold tolerance through ROS and autophagy and the photoprotection pathways. Here we summarize the latest findings on the function of BRs in the cold stress response and the regulatory networks that underpin it. We also review how BRs interact with other plant hormones in the cold stress response.

## 2. Brassinosteroid Signaling

**Figure 1:**
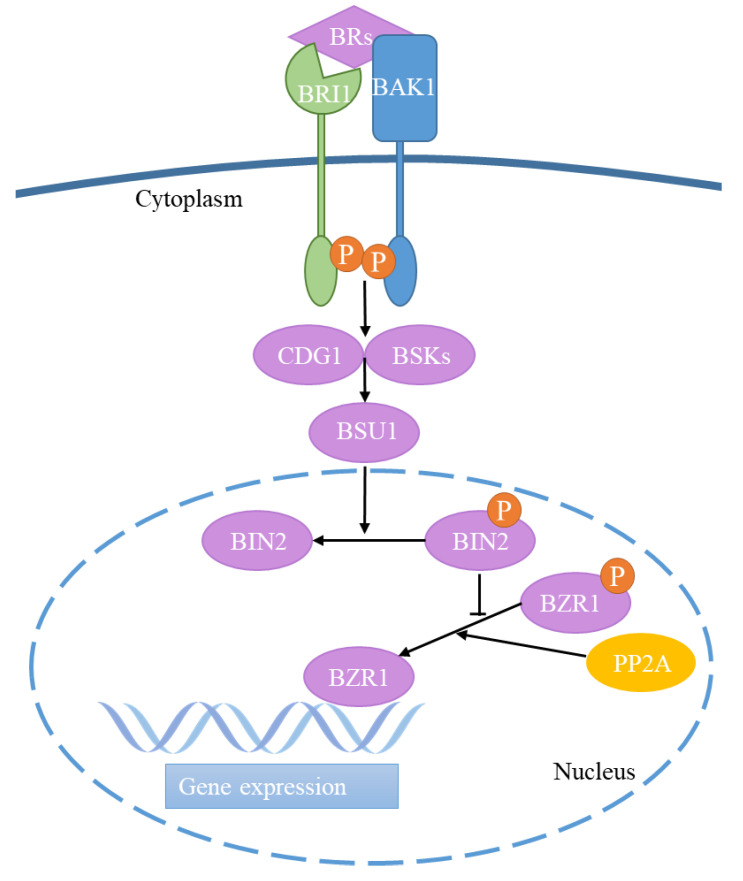
Model of BR signal transduction pathway. BRs bind to BRI1 and then phosphorylate with BAK1, and the phosphorylation of the two receptor kinases promotes the expression of BSKs and CDG1 families, which activate BSU1. Then, BSU1 inactivates BIN2 through dephosphorylation, thereby inhibiting the dephosphorylation of BZR1. PP2A also promotes the dephosphorylation of BZR1. Finally, non-phosphorylated BZR1 positively or negatively modulates hundreds of target genes. Abbreviations: BRs: brassinosteroids, BRI1: BRASSINOSTEROID INSENSITIVE1, BAK1: BRI1-ASSOCIATED KINASE1, BSKs: BR signaling kinases, CDG1: CONSTITUTIVE DIFFERENTIAL GROWTH1, BSU1: BRI1-SUPPRESSOR1, BIN2: BRASSINOSTEROID INSENSITIVE2, BZR1: BRASSINAZOLE-RESISTANT1, PP2A: protein phosphatase 2A, P: phosphorylates.

BRs are perceived by the extracellular structural domain of the cell surface receptor kinase BRASSINOSTEROID INSENSITIVE1 (BRI1), which then initiates a signal transduction cascade to regulate downstream gene transcription [8,16,17]. BRI1 activation involves the recruitment of the co-receptor kinase BRI1-ASSOCIATED KINASE1 (BAK1), and binding of extracellular domains of BRI1 and BAK1 causes the dissociation of BRI1 kinase inhibitor 1 (BKI1) from the cytoplasm membrane, which in turn leads to the activation of the kinase domains of BRI1 and BAK1 by mutual phosphorylation [18]. Post-activation BRI1 activates BR-SIGNALING KINASEs (BSKs) and CONSTITUTIVE DIFFERENTIAL GROWTH1 (CDG1) families through phosphorylation [19,20,21]. Then, BSKs and CDG1 activate the PP1-type phosphatase BRI1-SUPPRESSOR1 (BSU1), which subsequently dephosphorylates and inactivates BRASSINOSTEROID INSENSITIVE2 (BIN2) [18,20]. Activated BSU1 dephosphorylates the inactivating negative modulator BIN2, thereby relieving the phosphorylation inhibition of BIN2 on downstream transcription factors BRASSINAZOLE-RESISTANT1 (BZR1) and BRI1-EMS-SUPPRESSOR1 (BES1) [22,23]. Additionally, BRs recruit cytosolic phosphorylated BZR1 to the nucleus, where BZR1 then interacts with protein phosphatase 2A (PP2A) and is dephosphorylated by PP2A [24,25]. Non-phosphorylated BZR1/BES1 in the nucleus positively or negatively modulates hundreds of target genes [9,26] (Figure 1). Studies also found that BZR1/BES1 interacts with a variety of transcription factors. BZR1/BES1 interacts with BES1-interacting MYC-like 1 (BIM1) and MYB DOMAIN PROTEIN30 (MYB30) to regulate BR signaling in plants [27,28]. BZR1/BES1 interacts with the basic helix–loop–helix (bHLH) family of transcription factors, or the phytochrome interacting factors (PIFs), to mediate BR-regulated photomorphogenesis [29].

## 3. Brassinosteroids Enhance Plant Cold Resistance

Endogenous BR content rises in plants during cold stress. Brassinolide (BL), castorosterone (CS), and 28-norCS were the three detectable BRs in tomato (*Solanum lycopersicum* L.) leaves that showed an increase in levels when exposed to 8 °C for 8 h [30]. Conversely, the level of expression of the BR biosynthesis genes (*Cs90A1* and *Cs90B1*) in cucumber (*Cucumis sativus* L.) significantly decreased at low temperatures [31]. However, it has not been demonstrated whether the reduced expression of BR biosynthesis genes affects the BR content and the role of BRs in cold stress [32].

BR signaling is an important pathway to cold tolerance in plants. The study of receptor kinases and transcription factors for BR signaling revealed that overexpression of positive BR signaling components increases cold tolerance, whereas overexpression of negative components reduced cold tolerance. In cucumber, cold treatment significantly increased the positive regulators of BR signaling, *CsBES2*, *CsBES3*, *CsBIN1*, *CsBIN2*, and *CsBRI1* genes, and spraying exogenous 24-epibrassinolide (EBR) at low temperatures further increased the expression of the *CsBES1*, *CsBES2*, and *CsBES3* genes [31]. In tomato, compared with the wild-type (WT) plant, *bzr1* mutant plants had less cold tolerance [30]. In Arabidopsis, overexpression of *BRI1* significantly improved plant survival under cold stress, while the survival of *bri1-1* and *bri1-301* mutant plants was significantly reduced [33]. BIN2 acts as a negative component in the BR pathway. The triple mutant plants of *BIN2*, *bin2-3 bil1 bil2*, showed increased cold resistance, while the overexpression in *BIN2* plants caused decreased cold resistance [34].

In addition to directly changing the BR signal transduction components to enhance cold resistance, there are other methods that can regulate BR signaling transduction components to change cold tolerance of plants.

The G protein heterotrimer is a core component of plant signaling transduction and is composed of the Gα, Gβ, and Gγ subunits [35]. The G protein α subunit (GPA) plays an important role in a variety of signaling pathways, with specific functions in morphogenesis and abiotic stresses [36]. The G protein α subunit 1 (GPA1) can interact with the key BR signaling transduction gene, CDL1 [37]. In cucumber, the cold tolerance of *CsGPA*1- RNA interference (CsGPA1-RNAi) lines was significantly decreased, and the expression of *CsBZR1* and *CsBZR2* was significantly reduced in the *CsGPA1*-RNAi line at low temperatures. With prolonged low-temperature treatment, the expression levels of most BR-related genes were significantly decreased compared with WT, suggesting that CsGPA1 may enhance cold tolerance in plants by regulating BR synthesis and signaling gene expression [38]. The BASIC PENTACYSTEINE (BPC) proteins play an important role in regulating plant development and stress response [39]. In cucumber, *CsBPC2* mutant plants showed reduced cold tolerance and down-regulated expression of BR synthesis and BR signaling transduction genes, such as *CsBZR1* and *CsBZR2*, suggesting that BPC is involved in regulating BR synthesis and signaling to modulate the response to low temperatures [40].

Plants initiate a cascade of intracellular reactions to increase the content of BRs, which has a positive impact on the cold tolerance of plants. In tomato, overexpression of the BR biosynthesis gene DWARF (DWF: OX2) increased the content of endogenous BRs, whereas partial loss of function of DWARF in the dim mutant reduced the level of endogenous BRs [41]. After 3 days of cold stress at 4 °C, oxidized protein accumulation was significantly higher in dim plants compared with wild-type plants, whereas it was lower in DWF:OX2 plants. In addition, electrolyte leakage increased by 25.4% in dim plants compared with wild-type plants, whereas it decreased by 16% in DWF:OX2 plants [41].

Studies found that exogenous application of EBR increased freezing tolerance in Arabidopsis, tomato, and cucumber [41,42,43]. EBR regulates cold stress in phosphoproteome and proteome levels. EBR altered the response of cucumber to cold stress by modulating phosphorylation and proteomic levels, mainly through negative regulation of cold-responsive phosphopeptides and proteins. EBR up-regulated 16 cold-up-regulated phosphopeptides related to photosynthesis and nucleotide binding and also increased the expression of basic leucine zip (bZIP) phosphorylation levels and expression of hormone signal transduction genes. Most plant hormone genes related to growth, such as indoleacetic acid (IAA), cytokinin, and gibberellin (GA), were significantly down-regulated, whereas those associated with abiotic stress, such as BRs, jasmonic acid (JA), and abscisic acid (ABA), were up-regulated [31].

The ability of BRs to enhance cold tolerance is also reflected in their ability to extend the storage time of fruits by reducing the degree of cold injury (CI) suffered during storage at low temperatures. Studies have shown that in mango (*Mangifera indica* L.) the application of BL to mango fruit during low-temperature storage of 5 °C increased the degree of unsaturation and fluidity of the plasma membrane and reduced the susceptibility to membrane damage at low temperatures, which results in a reduction of CI [44]. During low-temperature storage of peaches (*Prunus persica* (L.) Batsch), transient expression of PpBZR1 in the fruit led to an increase in sucrose levels, which had a protective effect on the cell membrane [45]. In tomato, overexpression of the BR biosynthesis gene *SlCYP90B3* (*DWARF*) improves chilling tolerance in fruit by increasing *SICBF1* expression and antioxidant enzyme activity [46].

**Figure 2:**
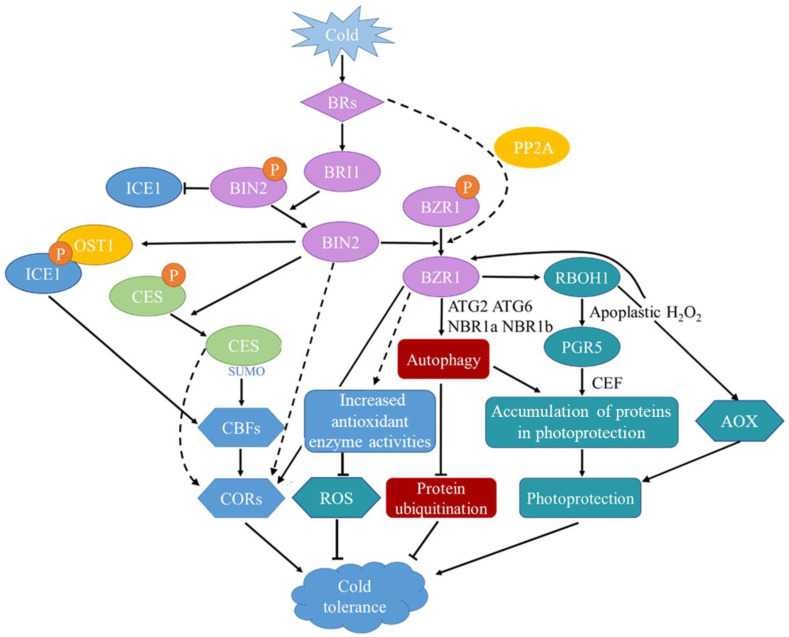
Model of BR regulation of cold stress response in plants. Under cold stress, plants regulate cold tolerance pathways through BR signaling, which can be divided into CBF-dependent and CBF-independent pathways. The CBF-dependent pathway primarily involves the dephosphorylation of the BR signaling inhibitor BIN2, leading to an increase in the transcriptional activity of ICE1. This in turn promotes the expression of CBFs and subsequently induces the expression of CORs, thereby enhancing cold tolerance. Conversely, the CBF-independent pathway is mediated by the dephosphorylation of BZR1 within the BR signaling cascade, which facilitates the expression of cold-related genes. This pathway enhances cold tolerance by increasing the activity of antioxidant enzymes to scavenge ROS, up-regulating the expression of autophagy-related genes to maintain cellular autophagic functions and prevent protein ubiquitination, and inducing the expression of PGR5 and AOX through the RBOH1 pathway to promote photoprotection under low-temperature conditions. Abbreviations: BRs: brassinosteroids, BRI1: BRASSINOSTEROID INSENSITIVE1, BIN2: BRASSINOSTEROID INSENSITIVE2, BZR1: BRASSINAZOLE-RESISTANT1, PP2A: protein phosphatase 2A, ICE1: INDUCER OF CBF EXPRESSION 1, OST1: OPEN STOMATA 1, CES: The BR-regulated bHLH TFs CESTA, CBF: C-repeat/DREB binding factor, COR: COLD-RESPONSIVE, ROS: reactive oxygen species, RBOH1: respiratory burst oxidase homolog 1, PGR5: PROTON GRADIENT REGULATION5, AOX: ALTERNATIVE OXIDASES, P: phosphorylates, SUMO: SUMOylation.

Cold induces the accumulation of C-repeat/DREB binding factor (CBF) proteins, which bind to the CRT/DRE motif in the *COLD-RESPONSIVE* (*COR*) genes and induce their expression [47]. Overexpression of *CBF* genes results in the induction of *COR* gene expression and increased freezing tolerance in many plant species [48]. In cold stress, BR signaling is regulated through the *CBF-COR* signaling pathway and DREB1/CBF-independent signaling pathways. At the same time, BRs can also enhance plant resistance to cold stress through *CBF*-independent signaling pathways. A simple model for BRs regulating cold stress response in plants is shown in Figure 2.

### 3.1. BRs’ Impact on CBF Regulation

The *CBF* gene is regulated by various transcription factors and signaling pathways, including BR signaling component genes. In tomato, the exogenous application of EBR significantly increased the values of the maximum quantum efficiency of photosystem II (*Fv/Fm)* and lowered the values of relative electrolyte leakage (REL) in the control plants; however, this effect was less in the pTRV-*CBF1* and pTRV-*CBF1/2/3* plants [49]. In *BRI1*-overexpressing *BRI-OE* plants, BR-signaling-deficient mutant *bri1-1* plants, and *bri1-301* plants, the expression of *CBFs* and downstream target genes was impaired in *bri1-1* plants and *bri1-301* plants. Meanwhile, *BRI1-OE* plants increased the basal levels of *CBFs*, in particular *CBF1* and *CBF3* [33].

The BR-regulated bHLH TFs CESTA (CES) and BZR1, which are the targets of *BIN2*, bind directly to *CBF* promoters and contribute to the regulation of constitutive *CBF* expression, in particular *CBF1* and *CBF3* [33]. During cold stress, dephosphorylated BZR1 accumulates in the nucleus and induces the expression cold-stress-related genes, whereas BIN2 negatively regulates cold resistance by inhibiting the dephosphorylation of BZR1 [34]. The expression of *CBFs* and their regulation were up-regulated in the triple mutant of BIN2 with its homologs *bin2-3 bil1 bil2* [50]. A BR-regulated bHLH TFs CESTA (CES) is the target of *BIN2* [51]. CES is the closest homolog of BRASSINOSTEROID ENHANCED EXPRESSION1 (BEE1) and BEE3 [52].

INDUCER OF CBF EXPRESSION 1 (ICE1) functions upstream of *CBFs* as a positive regulator. ICE1 is the substrate of BIN2, and BIN2 phosphorylating ICE1 promotes its degradation and reduces its transcriptional activity, which lowers the expression of *CBF* genes [50]. Early on in the cold stress response, BIN2 kinase activity is reduced and OPEN STOMATA 1 (OST1) kinase function is activated, coordinating the stabilization of ICE1 and stimulating maximal *CBF* gene expression. Meanwhile, with the increase in cold stress duration, the activity of BIN2 is increased and down-regulates the abundance of ICE1 after 3 h at 4 °C. Then, ICE1-regulated *CBF* expression is attenuated [50].

Compound sodium nitrophenolate (CSN) is a new artificially synthesized plant growth regulator that has been widely used in agricultural production on different crops [53]. In cucumber, we discovered that CSN improves cold tolerance by activating BR signaling, which leads to the expression of ICE-CBF-COR genes. Additionally, combining CSN with EBR enhances cold tolerance and aids in the recovery of cold-stressed seedlings [54].

### 3.2. BRs in CBF-Independent Signaling Pathways

BR regulation of plant cold tolerance acts largely in CBF-independent signaling pathways. Only approximately 10–25% of *COR* genes are regulated by *CBFs* [55,56]. In Arabidopsis, the CBF-independent regulatory pathway is mainly achieved through activation or inhibition of *COR* gene expression by BRI1 and CES [33].

*COR* genes uncoupled from CBFs under cold stress, including *PYR1-LIKE 6* (*PYL6*), *WRKY TF 6* (*WRKY6*), *SENESCENCE-ASSOCIATED GENE 21* (*SAG21*), *JASMONIC ACID CARBOXYL METHYLTRANSFERASE* (*JMT*), *SUPPRESSOR OF OVEREXPRESSION OF CO 1* (*SOC1*), and *EPITHIOSPECIFIER MODIFIER 1* (*ESM1*), have been identified to be regulated by BZR1. Additional ChIP and EMSA experiments revealed that BZR1 directly controls several of them [34].

The expression of relevant genes regulated by the CBF-dependent pathway in plants increased significantly during the early stages of cold stress and decreased during the later stages of stress [50]. Cold domestication is one of the main methods for plants to acquire cold tolerance, and the acquisition of cold domestication by plants seems to arise through CBF-independent pathways, such as the acquisition of cold stress memory and malondialdehyde (MDA) scavenging, as well as photoprotection and maintenance of autophagic functions, which are mostly directly or briefly associated with BZR1 [57].

#### 3.2.1. ROS

ROS are a class of byproducts in aerobic organisms, mainly including hydrogen peroxide (H_2_O_2_), superoxide anion radicals (O_2_^·−^), and hydroxyl radicals (·OH) [58]. ROS have a dual function. High concentrations lead to toxic secondary metabolites that can have a destructive effect on normal molecules in vivo; while low levels of ROS can be important as signaling molecules involved in the growth and development of organisms [59,60]. To eliminate ROS, plants produce antioxidant enzymes, including superoxide dismutase (SOD), catalase (CAT), peroxidase (POD), ascorbate peroxidase (APX), and glutathione reductase (GR) [61]. BRs are capable of eliminating ROS through the modification of antioxidant enzymes and non-enzymatic antioxidants [62]. In cucumber, exogenous application of EBR elevated antioxidant enzyme activities (SOD, POD, CAT, GR, APX) and decreased ROS (H_2_O_2_ and O_2_^·−^) levels in seedlings at low temperatures [63].

ROS can be generated by various enzymatic activities, the most well-studied of which are NADPH oxidases encoded by RESPIRATORY BURST OXIDASE HOMOLOGUE (RBOH), which are eliminated by a variety of ROS-scavenging enzymes [59]. NADPH oxidase is a redox enzyme that catalyzes the production of O_2_^·−^ from apoplast O_2_, accompanied by the production of H_2_O_2_, using cytoplasmic NADPH as an electron donor [64]. BRs increase the activity of ALTERNATIVE OXIDASES (AOX) in an RBOH-dependent manner [14]. Thus, a higher level of AOX regulates chloroplast and mitochondria’s electron flow via dissipating the excess energy, thereby decreasing ROS accumulation and increasing the protection of photosynthetic machinery [65]. Cold tolerance induced by BRs can be enhanced not only through the CBF pathway but also by increasing the activity of NADPH (RBOH) oxidase. This leads to an increase in H_2_O_2_ content, which acts as a signaling molecule to activate downstream photoprotective pathways, antioxidant enzyme systems, and other modulations under cold stress, thereby enhancing cold tolerance. This has been demonstrated in various plants such as pepper (*Capsicum annuum* L.), Arabidopsis, cucumber, and tomato [49,66,67]. Chilling stress produces ROS but treatment with EBR reduces the accumulation of H_2_O_2_ and superoxide anion, indicating that BRs can increase plant tolerance to low temperatures through crosstalk of reactive oxygen species. Under cold stress, EBR treatment significantly increased the activities of antioxidant enzymes in cucumber, while decreasing the contents of ROS and MDA [63]. In Arabidopsis, overexpression of the BR biosynthetic gene *Dwarf* or treatment with EBR attenuated chilling-induced oxidative damage [41]. By analyzing the upstream sequences of the *RBOH* family in Arabidopsis, tomato, rice (*Oryza sativa* L.), tobacco (*Nicotiana tabacum* L.), and cucumber, it was found that the BZR transcription factors of BRs have a broad binding range to the *RBOH* family promoters [57].

Plants respond to cold stress by maintaining their cold stress tolerance throughout recovery after cold stress acclimation (CS-ACC) [68]. We found that in cucumber, CS-ACC induced the acquisition of cold stress memory and enhanced the maintenance of acquired cold tolerance (MACT). Both BRs and RBOH-dependent signaling were necessary for the cold stress memory. These memory-responsive *CsRBOH*s and *CsBZR*s were not able to maintain their expression when RBOH-dependent signaling or BRs were inhibited [57].

BRs increased transcripts of *respiratory burst oxidase homolog 1 (RBOH1)* and *GLUTAREDOXIN* (*GRX*) genes, and BR-induced *RBOH1* and *GRX*s act as signaling components to enhance ROS scavenging capacity by regulating the activity of 2-Cys peroxiredoxin and other antioxidant enzymes [41]. Moreover, BZR1 binds to the conserved BRRE motifs of RBOH1’s promoters to positively up-regulate its expression. This in turn leads to RBOH1-dependent apoplast H_2_O_2_ generation, which in turn modifies the redox state to enhance BZR1 abundance, CBF gene transcription, and subsequently cold tolerance [49].

#### 3.2.2. Photosynthesis

Low temperatures will lead to the decrease in photosynthesis rate, chlorophyll content, and *Fv*/*Fm* of plants, leading to photoinhibition [68]. The degree of photoinhibition can be measured as a decrease in *Fv*/*Fm* or the maximal oxidation of P700 (DP700max) in photosystem I (PSI) [69]. To avoid photoinhibition caused by the overreduction of photosystems, plants have developed various photoprotective strategies, including leaf movements and chloroplast relocation to avoid light. These consist of the release of heat from absorbed light energy, cyclic electron flow (CEF) around photosystem II (PSI), photorespiratory and antioxidant pathways, and the repair cycle for damaged PSII (primarily the D1 protein) reaction centers [69,70,71]. Plants use photoprotection as a crucial defense mechanism against photoinhibition during stressful situations [70]. Research has demonstrated that in normal conditions, BRs regulate the Calvin cycle in a BZR1-dependent manner to enhance the ability of CO_2_ assimilation by raising gene expression and activating the enzymes involved in the Calvin cycle which in turn increases the photosynthetic rate [66,72,73].

In a low-temperature environment, exogenous EBR increased *Fv*/*Fm* and alleviated the decrease in photosynthesis caused by low temperatures [74]. In pepper, genes involved in photosynthesis were up-regulated by the application of exogenous EBR during cold stress, including eight encoding the PSI reaction center subunit, while ten genes encoding chlorophyll a/b-binding protein were also induced. Moreover, the genes involved in the ATP synthase chain, oxygen-evolving enhancer protein, ABC transporter I family members, thylakoid lumenal protein, and PsbP domain-containing protein were up-regulated. Induced by cold and BRs, BZR1 directly stimulates RBOH1 expression and apoplast H_2_O_2_ buildup [67]. Apoplast H_2_O_2_ is important for the induction of PROTON GRADIENT REGULATION5 (PGR5)-dependent cyclic electron flow [75,76]. PGR5 regulates chilling- and BR-induced non-photochemical quenching, D1, violaxanthinde-epoxidase (VDE), and PsbS accumulation, redox and hormone signaling, and antioxidant enzyme activity [77,78]. In tomato, mutations in BZR1 and PGR5, or with decreased RBOH1 transcription, compromised photoprotection provided by BR- and chilling-induced changes, making them more susceptible to photoinhibition. These findings show that, in response to cold stress, BRs function as a positive regulator of photoprotection in a redox-PGR5-dependent way [30].

BRs can also reduce photoinhibition after low temperatures and accelerate the recovery of photosynthetic apparatus from cold stress. In cucumber, exogenous EBR attenuated cold-induced PSII photoinhibition but no PSI photoinhibition during recovery in high light. EBR attenuated oxidative stress by increasing photosynthetic carbon fixation and antioxidant response and decreasing O_2_-dependent alternative electron flux [66]. These findings show that by activating Calvin cycle enzymes and improving antioxidant capacity, BRs hasten the recovery of the photosynthetic apparatus under high light conditions [66,79]. Therefore, under cold stress, BRs increase plant cold resistance by enhancing photoprotective capacity.

#### 3.2.3. Autophagy

Autophagy, a cellular recycling process, has multiple roles in eukaryotic organisms during differentiation, development, and stress responses [80]. Autophagy is a necessary mechanism for the degradation of unwanted and dysfunctional cells and is the main mechanism by which plants degrade macromolecular ubiquitinated protein aggregates in response to stress [80,81]. The process functions at a basal level under non-stress conditions and is highly induced by environmental stresses [82,83]. The autophagic machinery was initially identified in yeast, and the highly conserved *AUTOPHAGYRELATED* (*ATG*) genes encoding core components for autophagosome formation are also found in other organisms, including plants [84]. Today, the autophagy pathway is linked to more than 30 ATGs [80,85]. In plant responses to particular abiotic stressors, NEIGHBOR OF BRCA1 (NBR1), a selective autophagy receptor, facilitates the selective autophagosomal destruction of ubiquitinated protein aggregates [86,87]. Cold stress prevents the normal formation of autophagosomes and increases the ubiquitination level of insoluble proteins, making plants unable to degrade specific protein aggregates and organelles, which causes serious toxicity to plants and increases their susceptibility to low temperatures [82,88].

BR signaling can positively regulate autophagy under cold stress. In tomato, low temperatures and BRs increased the stability of BZR1, which can bind to the promoters of *ATG2*, *ATG6*, *NBR1a*, and *NBR1b* to activate their transcription and enhance autophagy. Autophagy inhibits NBR1-mediated ubiquitinated proteins by promoting the accumulation of photoprotective functional proteins (PsbS, VDE, and D1), which in turn improves cold tolerance [89].

## 4. Crosstalk between Brassinosteroids and Other Plant Hormones in the Cold Stress Response

Recent studies have shown that BRs work together with other plant hormones to regulate cold stress responses.

### 4.1. Crosstalk between BRs and Ethylene

Under low-temperature stress, exogenous application of EBR increases ABA, salicylic acid (SA), and JA content by 37.5%, 189.1%, and 132.3%, respectively, in pepper seedlings. However, EBR treatment down-regulated the expression of ethylene (ET) biosynthesis and signaling pathway genes. These genes included the ethylene biosynthesis gene *ACC synthase* (*ACS1*), the induction of the ethylene receptor (*ETR*), eight ethylene-responsive transcription factors, and ethylene insensitive 3-like 1 protein [67]. According to the findings, in pepper, EBR increased the enhanced endogenous levels of SA and JA while inhibiting ET biosynthesis in the regulation of cold response. In summary, BRs respond to low-temperature stress by synergistic crosstalk with auxin, JA, and SA and have an antagonistic relationship with ETH. BR signaling pathway activation and increased cold resistance are both facilitated by ETH.

However, in contrast to these findings, it has also been reported that BRs enhance ethylene biosynthesis in plants under cold stress. In cucumber, pretreatment by exogenous addition of 1 μm BL attenuated low-temperature-induced oxidative damage and significantly improved AOX and ethylene biosynthesis. In addition, BL treatment raised the transcript levels of genes involved in ethylene production. The application of salicylhydroxamic acid (SHAM, AOX inhibitor) and ethylene biosynthesis inhibitor blocked BR-induced alternate respiration and decreased plant resistance to cold stress, suggesting that in cucumber, ethylene is involved in BR-induced AOX activity to improve cucumber tolerance in cold stress [90].

These variations might be explained by the different studies’ use of different growing conditions or experimental designs. Further research is required to further our understanding of how ethylene and BRs interact to affect how various plant species respond to cold stress.

### 4.2. Crosstalk between BRs and ABA

ABA delays and slows down plant growth but also raises plant survival and resilience to different abiotic stresses including cold [91]. The complex antagonism between ABA and BR signaling pathways has been widely documented. To improve stress responses and adapt to adverse growth circumstances, the ABA receptors bind Abelson interactor 1 (ABI1) and Abelson interactor 2 (ABI2) to remove their inhibition on the kinase activity of BIN2, which results in a reduction of BR signaling outputs and higher ABA signaling outputs [92]. However, it remains unclear whether and how ABA synergizes with BRs in plants under cold stress. The ABA content in two tomato varieties significantly increased in response to low-temperature stress. A sharp increase in the ABA content was observed in cold-sensitive species that received EBR treatment compared with the control under low-temperature stress [93]. Recent reports have shown that BR-induced cold tolerance is dependent on ABA biosynthesis. In tomato, the cold-induced increase in endogenous BRs inhibits BIN2 abundance, leading to activation of BZR1, which promotes ABA biosynthesis by regulating the expression of the ABA biosynthesis gene *9-CIS-EPOXYCAROTENOID DIOXYGENASE1* (*NCED1*) [94].

### 4.3. Crosstalk between BRs and JA

The crosstalk between BRs and JA is critical in responding to abiotic stress. In Arabidopsis, cold stress induces the synthesis of JA, which then enhances cold tolerance by regulating both the ICE-CBF pathway and the CBF-independent pathway [95]. The synthesis of JA is induced by cold stress, and JA then favorably affects the freezing tolerance of Arabidopsis by controlling both the ICE-CBF route and the CBF-independent pathway. BES interacting BIM1 is a bHLH protein involved in brassinosteroid signaling [96]. In apple (*Malus × domestica*), BIM1 cannot only directly activate the transcription of *CBF1* but also form a complex with *CBF2* to enhance the transcriptional activation of corresponding *COR* genes, thus improving cold tolerance mediated by BR signaling. Two repressors of JA signaling, JAZMONATE ZIM-DOMAIN1 (JAZ1) and JAZMONATE ZIM-DOMAIN2 (JAZ2), integrate BRs and JA signaling by competition with BIM1 for binding to CBF2 to inhibit the BIM1–CBF2 heterodimeric complex formation. Meanwhile, JAZ1 and JAZ2 did not seem to have a substantial effect on BIM1 protein abundance. The parallel regulation module composed of BR-BIM1-CBF and JA-JAZ-BIM1-CBF ensures the efficient and timely response of plants to cold stress [97].

## 5. Concluding Remarks and Future Perspectives

The roles of BR signaling in fruit resistance to cold stress and plant growth and development have been thoroughly investigated by researchers in recent years, and they have progressively uncovered the relationships between BR signaling and other plant hormone signaling pathways. We anticipate that more regulatory variables related to the cold stress response within the BR regulatory network will become visible and characterized in the future to enhance plant characteristics. Even if some positive outcomes have been attained, there are still some unsolved issues that require further research to address: (1) Most research on BRs and BR signaling in response to cold stress is focused on model plants, such tomato and Arabidopsis. As a result, more crops need to be examined to confirm the obtained results. (2) Most of the studies on the effects of BRs and BR signaling on cold stress are focused on the seedling stage of crops. Fewer studies have been conducted on the reproductive growth stage, which is a critical period for crop yields. It is still unknown whether the effects of BRs on cold stress differ at different periods of plant growth and development. (3) Cold domestication is one of the important ways for plants to acquire low-temperature resistance, and BRs are involved in regulating this process, but the molecular mechanism remains to be further refined. (4) In terms of the crosstalk between BRs and other plant hormones, there appear to be different interactions among different plant species. Future studies are needed to further understand the complex relationships among hormones. In addition, the excavation of BR target genes that can simultaneously improve crop growth, tolerance, and resistance will be the focus of future work.
